# Identification and quantitative mRNA analysis of a novel splice variant of *GPIHBP1* in dairy cattle

**DOI:** 10.1186/2049-1891-5-50

**Published:** 2014-11-05

**Authors:** Jie Yang, Xuan Liu, Qin Zhang, Li Jiang

**Affiliations:** National Engineering Laboratory for Animal Breeding; Key Laboratory of Animal Genetics, Breeding and Reproduction, Ministry of Agriculture of China; College of Animal Science and Technology, China Agricultural University, Beijing, 100193 China

**Keywords:** Alternative splice variant, Cattle, Expression pattern, *GPIHBP1*

## Abstract

**Background:**

Identification of functional genes affecting milk production traits is very crucial for improving breeding efficiency in dairy cattle. Many potential candidate genes have been identified through our previous genome wide association study (GWAS). Of these, *GPIHBP1* is an important novel candidate gene for milk production traits. However, the mRNA structure of the bovine *GPIHBP1* gene is not fully determined up to now.

**Results:**

In this study, we identified a novel alternatively splice transcript variant (X5) which leads to a 31 bp insertion in exon 3 and also confirmed the other four existed transcripts (X1, X2, X3 and X4) of the bovine *GPIHBP1* gene. We showed that transcript X5 with a 31 bp insertion and transcript X1 with an 8 bp deletion might have tremendous effect on the protein function and structure of *GPIHBP1*, respectively. With semi-quantitative PCR and quantitative real-time RT-PCR, we found that the mRNA expression of *GPIHBP1*, *GPIHBP1*-X1 and *GPIHBP1*-X5 in mammary gland of lactating cows were much higher than that in other tissues.

**Conclusions:**

Our study reports a novel alternative splicing of *GPIHBP1* in bovine for the first time and provide useful information for the further functional analyses of *GPIHBP1* in dairy cattle.

**Electronic supplementary material:**

The online version of this article (doi:10.1186/2049-1891-5-50) contains supplementary material, which is available to authorized users.

## Background

Our previous genome-wide association study (GWAS) in Chinese Holstein population revealed Glycosylphosphatidylinositol-anchored HDL binding protein1 (*GPIHBP1*) is a potential candidate functional gene for milk production traits [1]. A SNP which is located 1,295 bp upstream from the translation initiation site of *GPIHBP1* gene showed strong association with milk yield trait, protein yield and fat percentage with *P* values 1.02E-10, 1.55E-07 and 6.30E-20, respectively. To confirm the association between the *GPIHBP1* gene and milk production traits, we selected a SNP within 5′UTR of *GPIHBP1* in another Chinese Holstein population for further association study. This SNP also showed very significant association with milk yield trait, fat percent trait and protein yield trait (unpublished data). Therefore, *GPIHBP1* was considered as a novel promising candidate functional gene in dairy cattle.

The GPIHBP1 protein is a glycosylphosphatidylinositol (GPI)-anchored protein of the lymphocyte antigen 6 family. It contains an N-terminal signal peptide, an acidic domain, a lymphocyte antigen 6 (Ly6) domain, and a hydrophobic carboxyl-terminal motif [2]. In the endoplasmic reticulum, the signal peptide is removed and the carboxyl-terminal hydrophobic sequence is replaced by a GPI-anchor [3]. Thus, the acidic domain and Ly6 motif are of great importance for mature GPIHBP1. Recent studies showed that they play an important role in the capacity of GPIHBP1 to bind lipoprotein lipase (LPL) [4]. It has been demonstrated that some mutations, such as C65Y, C89F and Q115P, in the most highly conserved portion of the Ly6 domain lead to the abolishment of GPIHBP1 to bind LPL [5–7], and a mutation in the C-terminal hydrophobic domain, G175R, markedly reduces the ability of GPIHBP1 to reach the cell membrane and bind LPL [7].

*GPIHBP1* is responsible for actively transporting LPL across endothelial cells [8]. Once inside capillaries, LPL hydrolyzes the triglycerides in plasma lipoproteins and provides the lipids from blood for production of milk lipids [9, 10]. Thus, *GPIHBP1* plays a critical role in the lipolytic processing of triglyceride-rich lipoproteins. Rios *et al.*[11] found that in human a deletion of 17.5 Kb containing the entire *GPIHBP1* gene resulted in extremely high plasma triglyceride and cholesterol level. Beigneux *et al.*[12] reported that glycosylation of Asn-76 within the Ly6 domain of the mouse GPIHBP1 was critical for its appearance on the cell surface. Beigneux *et al.*[12] showed the *GPIHBP1*-knockout (*GPIHBP1*-/-) mice displayed severe hypertriglyceridemia, with a plasma triglyceride level of 1,000-6,000 mg/dL at 7-10 week of age. It was reported that *GPIHBP1* was highly expressed in heart and adipose tissue in mice [12, 13] and its tissue expression pattern was similar to that of *LPL*[13]. Recent studies showed that *GPIHBP1* was the key element for transport and localization of *LPL*[8, 14, 15] and might serve as a platform for lipolysis on endothelial cells [3, 16].

Up to now, the genomic organization of *GPIHBP1* remains undetermined yet. The mRNA structure of the bovine *GPIHBP1* gene has been keeping on changing in the NCBI database in the most recent years. In the present study, we investigated a new splice variant of bovine *GPIHBP1*. In order to layout the groundwork for its biological function validation in dairy cattle, we also performed quantitative analysis of the mRNA expression patterns of *GPIHBP1* and its novel splice variant in different tissues. We aimed to establish which splice variant is predominantly expressed in bovine tissues.

## Methods

### Animals and tissue sample collection

Three Chinese Holstein cows which were in the same period of lactation were selected from Beijing Sanyuan Dairy Farm Center. All of them were fed in a consistent environmental condition. Eight tissues samples (heart, liver, lung, kidney, mammary gland, ovary, uterus and muscle) from each cow were collected within 30 min after slaughter and stored at liquid nitrogen. The whole procedure for collection of the tissue samples of all animals was carried out in strict accordance with the protocol approved by the Animal Welfare Committee of China Agricultural University (Permit number: DK996).

### RNA extraction and reverse transcription

The total RNA was extracted from the eight tissues of the three cows by using Trizol reagent (Invitrogen, CA, USA). The quantity and quality of RNA were measured via an ND-2000 spectrophotometer (Thermo, USA). Reverse transcription (RT) was carried out in a solution of 20 μL, containing 12 μL Mix (0.5 μL Primer (50 μmol/L) oligio(dt), 0.5 μL Random primer, 1 μL dNTPs (10 mmol/L), 5 μg total RNA and ddH_2_O up to 12 μL), 4 μL 5 × First-Strand buffer, 2 μL 0.1 mol/L dTT, 1 μL RNaseout (40U/μL), and 1 μL SuperScrip III RT (200U/μL) (Life, USA). The Mix was heated at 65°C for 5 min and then incubated on ice for at least 1 min. Tubes containing all contents were incubated at 25°C for 5 min, 50°C for 60 min and 70°C for 15 min. To ensure the quality of the first strand cDNA, 1 μL of cDNA was used in a PCR reaction to amplify the glyceraldehyde phosphate dehydrogenase (*GAPDH*) gene.

### Polymerase chain reaction and clone sequencing

PCR reactions were performed to amplify the coding regions of *GPIHBP1*. The primers (Primer1, Primer2 and Primer3, Additional file 1: Table S1) were designed using the Primer 3 web-tool (http://frodo.wi.mit.edu/primer3/) and the Oligo 6.0 software. For each amplicon, 1 μL of cDNA (1,000 ng/μL), 2.0 μL of 10× PCR buffer, 250 μmol/L of each dNTP, 0.5 units of HotstarTaq polymerase (Takara Biotechnology, Tokyo, Japan), and 0.5 μmol/L of primer (Life Technologies) were used in a total 20 μL reaction. The reaction was denatured for 10 min at 95°C, then 35 cycles of 94°C for 30 s, special annealing temperature for 30 s and 72°C for 30 s, and a final extension of 72°C for ten min. The products were electrophoresed on 2% agarose gels and stained with ethidium bromide.

The purified double-stranded DNA (Omega, USA) was cloned in pMD18-T (Takara Biotechnology, Tokyo, Japan). The products of the ligation reactions were transformed into competent cells. Twenty colonies per sample were selected randomly for sequencing. With the DNAMAN 7.0 software, we performed multiple sequences alignment analysis.

### Predicted structures of the GPIHBP1 protein

The T coffee website tool (http://tcoffee.vital-it.ch/apps/tcoffee/do:regular) was used to align amino acid sequences of the bovine and human GPIHBP1 proteins. We predicted the open reading frame of the bovine *GPIHBP1* transcript X5 using ORF Finder (http://www.ncbi.nlm.nih.gov/projects/gorf/). Secondary structures of the GPIHBP1 proteins were predicted using the PSIPRED v3.3 website tool (http://bioinf.cs.ucl.ac.uk/psipred/). SignalP 4.1 (http://www.cbs.dtu.dk/services/SignalP/) [17] was used to predict the presence and location of the signal peptide of GPIHBP1. Big-PI Predictor (http://mendel.imp.ac.at/gpi/gpi_server.html) [18] was utilized to predict GPI anchor sites in protein sequence. The human CD59 (membrane-bound glycoprotein) gene which also has the UPAR/Ly6 domain [19], was used as the reference for predicted bovine GPIHBP1 tertiary structures using the SWISS MODEL method (http://swissmodel.expasy.org/) [20]. The reported human CD59 (membrane-bound glycoprotein) served as the reference for predicted bovine GPIHBP1 tertiary structures using SWISS MODEL method (http://swissmodel.expasy.org/) [20].

### Real time RT-PCR

Real-time PCR (RT-PCR) was performed on the eight tissues of three cows. TaqMan Real-time PCR assays were performed using 7500Fast (Life, USA). The PCR amplification mix consisted of 2 μL 10× PCR Buffer, 1.2 μL Mg^2+^ (50 mmol/L), 0.5 μL dNTPs (10 mmol/L), 0.5 μL of each primer (10 μmol/L, Additional file 1: Table S2), 0.2 μL Taqman probe (GPI-Probe, X1-Probe and X5-Probe, 10 μmol/L, Additional file 1: Table S2), 1 μL cDNA, 0.2 μL Taq polymerase and 13.9 μL ddH_2_O in a final volume of 20 μL. The reaction was performed with the conditions as follows: an initial 2 min hold at 95°C, 50 cycles of 95°C for 10 s, 60°C for 30 s. The assays were carried out in triplicate and the average C_T_ values were obtained to calculate gene expression level. In addition, parallel assays using the same cDNA were carried out using the primers (Additional file 1: Table S2) and probe (*GPADH*-Probe, Additional file 1: Table S2) to the housekeeping gene *GAPDH*. The relative mRNA expression levels of *GPIHBP1* and two alternative splice variants were normalized to the *GAPDH* gene by the 2^-ΔΔCT^ method [21].

## Results

### Identification of a novel mRNA spliced variant of *GPIHBP1*

**Figure 1:**
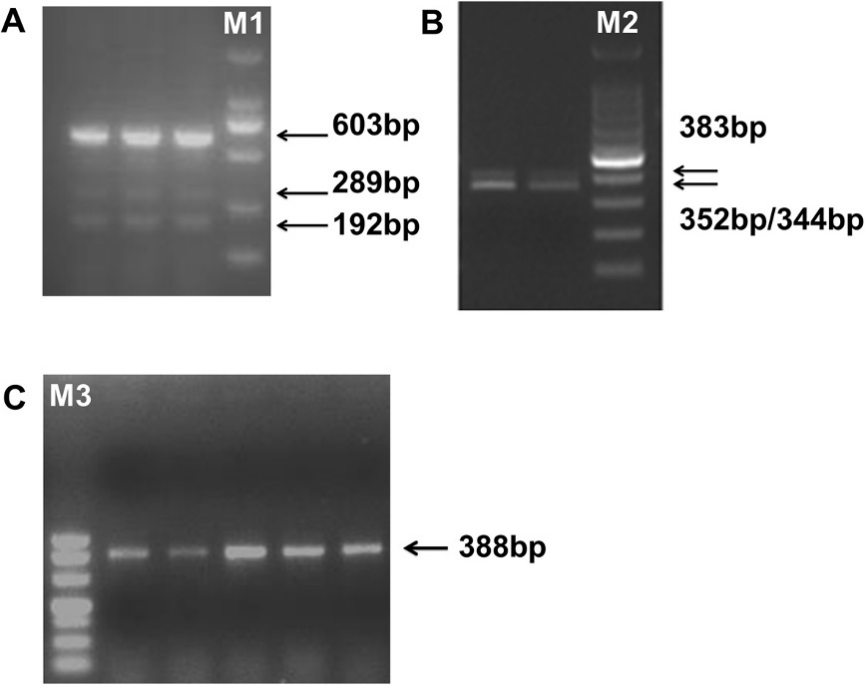
**PCR analysis of the** ***GPIHBP1*** **coding region in samples of mammary gland using three pairs of primers. (A)** With primer 1, transcript X2 (603 bp), transcript X3 (289 bp) and transcript X4 (192 bp) were observed as expected (according to the mRNA sequence of the bovine *GPIHBP1* gene in NCBI database). M1: DL2000, 2,000 bp, 1,000 bp, 750 bp, 500 bp, 250 bp, 100 bp. **(B)** With primer 2, in addition to the expected PCR band (352 bp and 344 bp), a band of 383 bp was also observed in all samples. M2: 100 bp DNA Ladder from 1,500–100 bp. **(C)** With primer 3, only the expected band (388 bp) was observed in all samples. M3: DL500, 500 bp, 400 bp, 300 bp, 200 bp, 150 bp, 100 bp and 50 bp.

Three primers (Additional file 1: Table S1) were used to amplify the coding region of *GPIHBP1* in samples of mammary gland. After PCR amplification with primer 1 and primer 3, we observed three (Figure 1A) and one (Figure 1C) PCR bands, respectively. These 4 bands correspond to the expected fragment size (Additional file 1: Table S1) derived from the bovine *GPIHBP1* sequence in the NCBI database. With primer 2, two bands with fragment lengths of 352 bp and 344 bp, respectively, would be expected according to bovine *GPIHBP1* sequence. However, the PCR products showed the two bands were almost merged into one band (Figure 1B), since there is only 8 bp difference in size between the two fragments. Interestingly, we observed an additional band of 383 bp which was present in all samples (Figure 1B, only two samples are shown). To verify the results of primer 2, we purified the PCR products and cloned them in Pmd18-T (Takara Biotechnology, Tokyo, Japan). We then randomly selected twenty colonies to sequence. It turned out that 17 of them were of 352 bp length, one of them showed a deletion of 8 bp (5′-GGCCGCAG-3′, Chr14:2550837-Chr14:2550830) in exon 3 and was of length of 344 bp, and two of them showed an insertion of 31 bp (5′-TGGAGGTTTACAGGTGTCCCTGCGCGGCCAG-3′, Chr14:2551602-Chr14:2551572) in exon 3 and were of length of 383 bp. Thus, both the two expected fragments and the novel fragment were confirmed.

**Figure 2:**
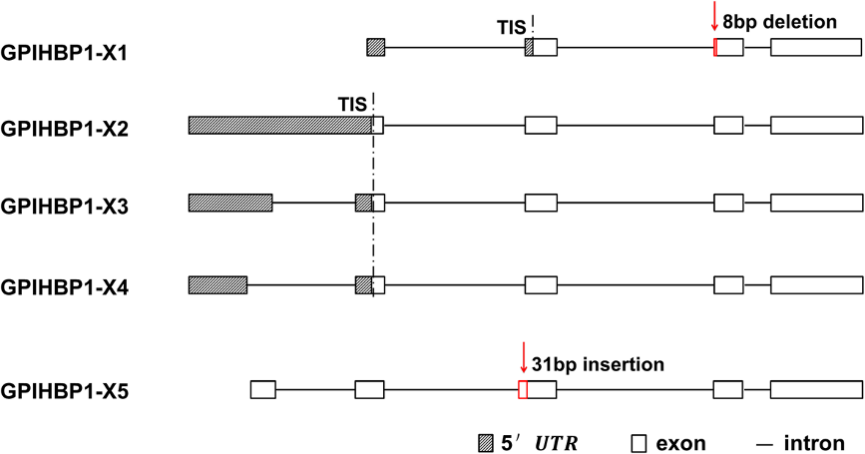
**Schematic representation of the** ***GPIHBP1*** **alternative splice variants.** Transcripts X1 (XM_002692563.2), X2 (XM_005215283.1), X3 (XM_005215284.1) and X4 (XM_005215285.1) were obtained from NCBI. Transcript X5 was observed by RNA-seq (unpublished data). Transcript X1 contained a 8 bp deletion in exon 3. Transcript X5 contained a 31 bp insertion in exon 3. Although transcript X2, X3 and X4 had different 5′ untranslated region, they had the same translation initiation site (TIS).

Currently, four transcript variants (X1, X2, X3 and X4) of *GPIHBP1* are presented in NCBI. Transcript X1, which leads to colonies with a deletion of 8 bp nucleotides, was reported very recently. However, the colonies with an insertion of 31 bp nucleotides suggest that there may exist a novel transcript variant in the bovine *GPIHBP1* gene. This novel transcript variant was named transcript variant X5 [GenBank accession number: KJ502292]. To further confirm that the 8 bp deletion and the 31 bp insertion were not a cloning artifact, we designed two pairs of primers (Additional file 1: Table S3) specifically for the 8 bp deletion and the 31 bp insertion sequences, respectively, and performed PCR amplification. As a result, we obtained a fragment of 207 bp for transcript X1 and a fragment of 101 bp for transcript X5. The existence of transcript variants X1 and X5 were thus confirmed again. The structures of different splice forms of *GPIHBP1* were shown in Figure 2. And there were notable differences in 5′ untranslated region (UTR) of different *GPIHBP1* transcripts. Out of five different splice forms, three (X2,X3 and X4) have the same translation initiation site.

### Characteristics of the *GPIHBP1*splice variants

Transcript variants X2, X3 and X4 have the same open reading frame (ORF) and encode a 171-amino acid protein that was named bovine GPIHBP1 P2. In contrast, the transcript variant X1 contains a different ORF and encodes a 142-amino acid protein, which named bovine GPIHBP1 P1. However, the ORF of transcript X5 was still not known clearly up to now. The ORF Finder software (http://www.ncbi.nlm.nih.gov/gorf/gorf.html) was used to predict all possible ORF of transcript variants X5. As a result, five potential ORF were predicted which had initiation codon and termination codon. The amino acid sequences corresponding to the five ORF were obtained using the DNAMAN 7.0 software and named bovine GPIHBP1 P5.1, P5.2, P5.3, P5.4, and P5.5, respectively (Additional file 1: Table S4).

### Predicted structures of the GPIHBP1 protein

**Figure 3:**
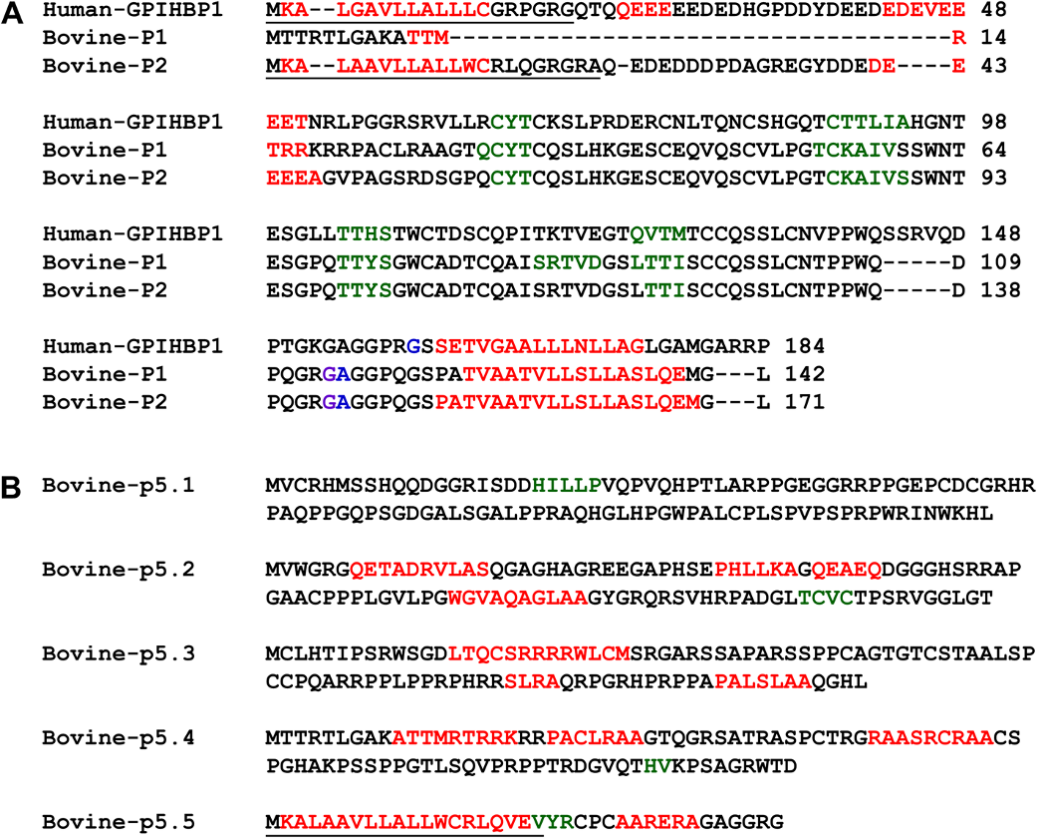
**Predicted secondary structures of bovine and human GPIHBP1.** Residues involved in the N-signal peptide formation are indicated by underline, *α*-helices are in red, *β*-sheets are in green, GPI-modification site is in blue, alternative GPI-modification site is in purple. **(A)** Amino acid sequence alignments of bovine and human GPIHBP1. The predicted secondary structures for bovine-p1 (XP_002692609.2) and bovine-p2 (XP_005215340.1, XP_005215341.1 and XP_005215342.1) are similar for several regions with that of human GPIHBP1. **(B)** The predicted secondary structures for bovine-p5.1, bovine-p5.2, bovine-p5.3, bovine-p5.4 and bovine-p5.5 of transcript X5.

**Figure 4:**
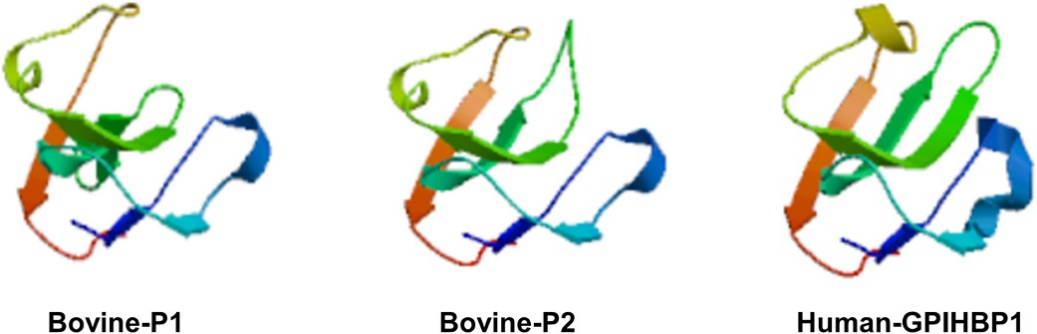
**Predicted tertiary structures of bovine and human GPIHBP1.** The reported human CD59 was used as the reference to obtain predicted GPIHBP1 tertiary structures by the SWISS MODEL method. The rainbow color code describes the tertiary structures from the N-termini (blue) to C-termini (red) for GPIHBP1 UPAR/Ly6 domains. Arrows indicate the directions for *β*-sheets.

The predicted secondary structures of the bovine GPIHBP1 amino acid sequences were compared with that of the human GPIHBP1 protein. The *α*-Helix and *β*-sheet structures of bovine GPIHBP1 P1 and P2 were similar to the human GPIHBP1 protein secondary structure (Figure 3A). By using SignalP 4.1 [17], we found that only human GPIHBP1, bovine GPIHBP1 P2 and bovine GPIHBP1 P5.5 had N-terminal signal peptide which contained a predicted helical structure (Figure 3A, B). The GPI-modification sites of the human GPIHBP1 protein, bovine GPIHBP1 P1 and bovine GPIHBP1 P2 were also predicted (*P* < 0.01) using Big-PI Predictor [18, 22] (Additional file 1: Table S5). We found that bovine GPIHBP1 P1 and P2 and the human GPIHBP1 protein had similar position for alternative GPI-modification site. The predicted tertiary structures of the bovine GPIHBP1 P1, bovine GPIHBP1 P2 and human GPIHBP1 sequences are shown in Figure 4. It can be seen that these tertiary structures were similar to the reported UPAR-LY6 domain of the human CD59 protein [19]. Their modeling ranges of residues were 62-138aa, 28-104aa and 58-133aa, respectively, which contained four or five *β*-sheet structures. However, due to the low alignment quality between target and specified template, the tertiary structures of the predicted bovine GPIHBP1 P5.1, P5.2, P5.3, P5.4, and P5.5 could not be constructed.

### Tissue mRNA expression pattern of three splice variants of *GPIHBP1*

It can be seen from Figure 2 that the difference in 5′ untranslated region (UTR) of *GPIHBP1* transcripts X2, X3 and X4 did not affect the structure of protein. In contrast, the 8 bp deletion of transcript X1 and 31 bp insertion of transcript X5 had tremendous effect on the structure and function of protein. Thus, semi-quantitative PCR and TaqMan Real-time PCR were employed with specific primers for the 8 bp deletion of transcript X1, 31 bp insertion of transcript X5 and overall GPIHBP1 transcripts in eight tissues of three cows.

**Figure 5:**
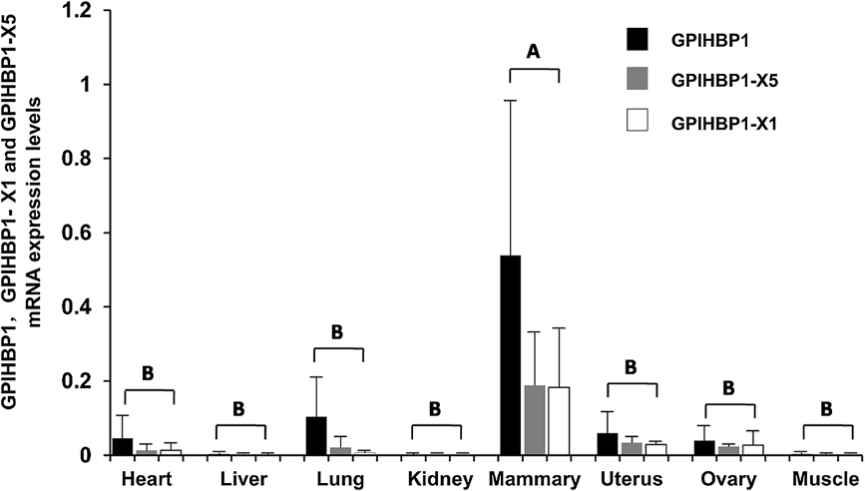
**The mRNA expression patterns of** ***GPIHBP1*** **,** ***GPIHBP1*** **-X1 and** ***GPIHBP1*** **-X5 revealed by RT-PCR.** The histograms represent the mRNA expression level of *GPIHBP1*, *GPIHBP1*-X1 and *GPIHBP1*-X5 in eight tissues of three cows. mRNA expression levels in mammary gland of *GPIHBP1*, *GPIHBP1*-X1 and *GPIHBP1*-X5 all were highest among 8 tissues. The different capital letters indicated significant differences in the expression among eight tissues at *P* <0.01.

TaqMan Real-time PCR analysis was conducted to further identify the tissue mRNA expression pattern of bovine *GPIHBP1*. After normalization with the corresponding mRNA expression level of the housekeeping gene *GAPDH*, analysis of variance (ANOVA) and multiple comparisons were conducted with R software. We found that mRNA expression level of *GPIHBP1*, *GPIHBP1*-X1 and *GPIHBP1*-X5 all were significantly different among eight tissues (*P* < 0.05), in which the mRNA expression levels were significantly higher in mammary gland than other tissues (*P* < 0.05). And all of *GPIHBP1*-X1, *GPIHBP1*-X5 and overall *GPIHBP1* had much lower expression level in liver, kidney and muscle (Figure 5).

## Discussion

Tissue-specific mRNA expression patterns are important for revealing functional candidate genes associated with milk production traits [23]. The specifically high expression of *GPIHBP1* in mammary gland suggests that it may play an important role in milk production traits or mammogenesis. It has been reported that *GPIHBP1* was highly expressed in mammary fat and heart tissues in mice [12, 13]. Previous studies [24–26] showed that the lipoprotein lipase-mediated processing of lipoproteins within mammary gland is important for providing the lipid nutrients to produce milk fat. And the lipoprotein lipase (*LPL*) expression pattern in bovine mammary gland at different stages of lactation was quite similar to the lactation curve, which suggest that *LPL* is important for maintenance of milk synthesis [27]. Meanwhile, some studies on hyperlipidemia showed that *GPIHBP1* served as the transporter and the platform for the lipoprotein lipase-mediated lipolysis processing [28]. Therefore, *GPIHBP1* is essential for LPL to realize its biological function and play an important role in the process of producing milk fat and maintenance of milk synthesis.

Commonly, alternative splicing may change the structure of transcripts of a gene and the protein encoded by the gene, leading to profound functional alternation. It has been demonstrated that alternative splicing could affect the binding properties, intracellular localization, enzymatic activity, protein stability and post-translational modifications of a large number of proteins [29]. The effects of alternative splicing range from complete loss of function or gain of a new function to very subtle modulations that are difficult to detect [30]. Changes in alternative splicing of a gene can modulate its mRNA expression levels by subjecting mRNAs to nonsense-mediated decay (NMD) and alter the structure of protein [30]. Alternative splicing is regulated by splicing codes, including exonic splicing enhancers (ESEs), exonic splicing silencers (ESSs), intronic splicing enhancers (ISEs) and intronic splicing silencers (ISSs) [31]. Tissue-specific mRNA expression pattern could be associated with absence or presence of splicing codes in various tissues.

In this study, we identified that there were five transcripts (X1, X2, X3, X4 and X5) of the bovine *GPIHBP1* gene. The proteins of transcripts X2, X3 and X4 have the classical structure of the GPIHBP1 protein consisting of the N-terminal signal peptide, UPAR-Ly6 domain and C-terminal GPI-Modification Site. The protein P1 encoded by transcript X1 has the UPAR-Ly6 domain and the C-terminal GPI-Modification Site, but it lacks the signal peptide and acidic domain. It is not clear if this protein would be ever produced and secreted because of lacking the signal peptide. However, even if it is at all secreted as normal, it is also a non-functional GPIHBP1 because it lacks acidic domain, which makes it unable to bind to LPL [32]. The splicing resulting in the transcript X5 has a tremendous effect on the protein structure. The predicted secondary structures of bovine GPIHBP1 P5.1, P5.2, P5.3, P5.4 and P5.5 are quite different from that of bovine GPIHBP1 P1, P2 and human GPIHBP1 (Figure 3B). They do not have the UPAR-LY6 domain, which is considered as a very important functional region of GPIHBP1 [32]. Therefore, this novel splicing variant may regulate the transcript abundance of *GPIHBP1* in mammary gland of dairy cattle by nonsense-mediated decay and thus affect milk production traits indirectly.

## Conclusions

This study is the first report on alternative splicing of bovine *GPIHBP1* gene. We identified a novel alternatively spliced transcript variant of *GPIHBP1* gene (*GPIHBP1*-X5) with 31 bp insertion in the exon and also confirmed other four existed transcripts (X1, X2, X3 and X4) of the *GPIHBP1* in Chinese Holstein cow. And we found that the 8 bp deletion of transcript X1 and 31 bp insertion of transcript X5 have tremendous effect on protein function and structure, respectively. Based on the results of Taq-Man RT-PCR, we found that *GPIHBP1*-X1, *GPIHBP1*-X5 and *GPIHBP1* expressed in higher level in mammary gland than in other tissues of lactating dairy cow. In conclusions, our findings provided more information for the further functional analyses of *GPIHBP1* in dairy cattle.

## Electronic supplementary material

Additional file 1: Table S1: Sequences of forward (F) and reverse (R) primers for PCR amplification of the coding region of GPIHBP1. **Table S2.** Sequences of forward (F) and reverse (R) primers and probes used for TaqMan real time PCR. **Table S3.** Specific primers for confirming the existence of 8 bp deletion and 31 bp insertion. **Table S4.** The amino acid sequences of five potential open reading frames of GPIHBP1 X5. **Table S5.** Predicted GPI-modification sites for amino acid sequence of GPIHBP1 splice variants. (XLSX 15 KB)

## Abbreviations

GPIHBP1: Glycosylphosphatidylinositol-anchored HDL binding protein1
LPL: Lipoprotein lipase
UTR: Untranslated region
ORF: Open reading frame
NMD: Nonsense-mediated decay
ESEs: Exonic splicing enhancers
ESSs: Exonic splicing silencers
ISEs: Intronic splicing enhancers
ISSs: Intronic splicing silencers.

## References

[1] Jiang L, Liu J, Sun D, Ma P, Ding X, Yu Y, Zhang Q (2010). Genome wide association studies for milk production traits in Chinese Holstein population. PLoS One.

[2] Beigneux AP, Davies BS, Bensadoun A, Fong LG, Young SG (2009). GPIHBP1, a GPI-anchored protein required for the lipolytic processing of triglyceride-rich lipoproteins. J Lipid Res.

[3] Young SG, Davies BS, Fong LG, Gin P, Weinstein MM, Bensadoun A, Beigneux AP (2007). GPIHBP1: an endothelial cell molecule important for the lipolytic processing of chylomicrons. Curr Opin Lipidol.

[4] Gin P, Yin L, Davies BS, Weinstein MM, Ryan RO, Bensadoun A, Fong LG, Young SG, Beigneux AP (2008). The acidic domain of GPIHBP1 is important for the binding of lipoprotein lipase and chylomicrons. J Biol Chem.

[5] Franssen R, Young SG, Peelman F, Hertecant J, Sierts JA, Schimmel AW, Bensadoun A, Kastelein JJ, Fong LG, Dallinga-Thie GM, Beigneux AP (2010). Chylomicronemia with low postheparin lipoprotein lipase levels in the setting of GPIHBP1 defects. Circ Cardiovasc Genet.

[6] Beigneux AP, Franssen R, Bensadoun A, Gin P, Melford K, Peter J, Walzem RL, Weinstein MM, Davies BS, Kuivenhoven JA, Kastelein JJ, Fong LG, Dallinga-Thie GM, Young SG (2009). Chylomicronemia with a mutant GPIHBP1 (Q115P) that cannot bind lipoprotein lipase. Arterioscler Thromb Vasc Biol.

[7] Charriere S, Peretti N, Bernard S, Di Filippo M, Sassolas A, Merlin M, Delay M, Debard C, Lefai E, Lachaux A, Moulin P, Marcais C (2011). GPIHBP1 C89F neomutation and hydrophobic C-terminal domain G175R mutation in two pedigrees with severe hyperchylomicronemia. J Clin Endocrinol Metab.

[8] Davies BS, Beigneux AP, Barnes RH, Tu Y, Gin P, Weinstein MM, Nobumori C, Nyren R, Goldberg I, Olivecrona G, Bensadoun A, Young SG, Fong LG (2010). GPIHBP1 is responsible for the entry of lipoprotein lipase into capillaries. Cell Metab.

[9] Dallinga-Thie GM, Franssen R, Mooij HL, Visser ME, Hassing HC, Peelman F, Kastelein JJ, Peterfy M, Nieuwdorp M (2010). The metabolism of triglyceride-rich lipoproteins revisited: new players, new insight. Atherosclerosis.

[10] Beigneux AP, Davies BS, Tat S, Chen J, Gin P, Voss CV, Weinstein MM, Bensadoun A, Pullinger CR, Fong LG, Young SG (2011). Assessing the role of the glycosylphosphatidylinositol-anchored high density lipoprotein-binding protein 1 (GPIHBP1) three-finger domain in binding lipoprotein lipase. J Biol Chem.

[11] Rios JJ, Shastry S, Jasso J, Hauser N, Garg A, Bensadoun A, Cohen JC, Hobbs HH (2012). Deletion of GPIHBP1 causing severe chylomicronemia. J Inherit Metab Dis.

[12] Beigneux AP, Weinstein MM, Davies BS, Gin P, Bensadoun A, Fong LG, Young SG (2009). GPIHBP1 and lipolysis: an update. Curr Opin Lipidol.

[13] Beigneux AP, Davies BS, Gin P, Weinstein MM, Farber E, Qiao X, Peale F, Bunting S, Walzem RL, Wong JS, Blaner WS, Ding Z, Melford K, Wongsiriroj N, Shu X, de Sauvage F, Ryan R, Fong LG, Bensadoun A, Young SG (2007). Glycosylphosphatidylinositol-anchored high-density lipoprotein-binding protein 1 plays a critical role in the lipolytic processing of chylomicrons. Cell Metab.

[14] Davies BS, Goulbourne CN, Barnes RH, Turlo KA, Gin P, Vaughan S, Vaux DJ, Bensadoun A, Beigneux AP, Fong LG, Young SG (2012). Assessing mechanisms of GPIHBP1 and lipoprotein lipase movement across endothelial cells. J Lipid Res.

[15] Olivecrona G, Ehrenborg E, Semb H, Makoveichuk E, Lindberg A, Hayden MR, Gin P, Davies BS, Weinstein MM, Fong LG, Beigneux AP, Young SG, Olivecrona T, Hernell O (2010). Mutation of conserved cysteines in the Ly6 domain of GPIHBP1 in familial chylomicronemia. J Lipid Res.

[16] Young SG, Zechner R (2013). Biochemistry and pathophysiology of intravascular and intracellular lipolysis. Genes Dev.

[17] Petersen TN, Brunak S, Von Heijne G, Nielsen H (2011). SignalP 4.0: discriminating signal peptides from transmembrane regions. Nat Methods.

[18] Eisenhaber B, Bork P, Eisenhaber F (1999). Prediction of potential GPI-modification sites in proprotein sequences. J Mol Biol.

[19] Leath KJ, Johnson S, Roversi P, Hughes TR, Smith RA, Mackenzie L, Morgan BP, Lea SM (2007). High-resolution structures of bacterially expressed soluble human CD59. Acta Crystallogr Sect F: Struct Biol Cryst Commun.

[20] Arnold K, Bordoli L, Kopp J, Schwede T (2006). The SWISS-MODEL workspace: a web-based environment for protein structure homology modelling. Bioinformatics.

[21] Livak KJ, Schmittgen TD (2001). Analysis of relative gene expression data using real-time quantitative PCR and the 2(-Delta Delta C(T)) Method. Methods.

[22] Sunyaev SR, Eisenhaber F, Rodchenkov IV, Eisenhaber B, Tumanyan VG, Kuznetsov EN (1999). PSIC: profile extraction from sequence alignments with position-specific counts of independent observations. Protein Eng.

[23] Weikard R, Goldammer T, Brunner RM, Kuehn C (2012). Tissue-specific mRNA expression patterns reveal a coordinated metabolic response associated with genetic selection for milk production in cows. Physiol Genomics.

[24] Rudolph MC, McManaman JL, Phang T, Russell T, Kominsky DJ, Serkova NJ, Stein T, Anderson SM, Neville MC (2007). Metabolic regulation in the lactating mammary gland: a lipid synthesizing machine. Physiol Genomics.

[25] Jensen DR, Gavigan S, Sawicki V, Witsell DL, Eckel RH, Neville MC (1994). Regulation of lipoprotein lipase activity and mRNA in the mammary gland of the lactating mouse. Biochem J.

[26] Fisher EA (2010). GPIHBP1: lipoprotein lipase’s ticket to ride. Cell Metab.

[27] Bionaz M, Loor JJ (2008). Gene networks driving bovine milk fat synthesis during the lactation cycle. BMC Genomics.

[28] Adeyo O, Goulbourne CN, Bensadoun A, Beigneux AP, Fong LG, Young SG (2012). Glycosylphosphatidylinositol-anchored high-density lipoprotein-binding protein 1 and the intravascular processing of triglyceride-rich lipoproteins. J Intern Med.

[29] Kelemen O, Convertini P, Zhang Z, Wen Y, Shen M, Falaleeva M, Stamm S (2013). Function of alternative splicing. Gene.

[30] Stamm S, Ben-Ari S, Rafalska I, Tang Y, Zhang Z, Toiber D, Thanaraj TA, Soreq H (2005). Function of alternative splicing. Gene.

[31] Wang Z, Burge CB (2008). Splicing regulation: from a parts list of regulatory elements to an integrated splicing code. RNA.

[32] Beigneux AP, Gin P, Davies BS, Weinstein MM, Bensadoun A, Fong LG, Young SG (2009). Highly conserved cysteines within the Ly6 domain of GPIHBP1 are crucial for the binding of lipoprotein lipase. J Biol Chem.

